# 

**Published:** 1855-01

**Authors:** 


*Books Received.*—Harrison's Anatomy, the Dublin Dissector, Arink's Medical Manual, Horner's Naval Practice, and the Report of Hon. Hamilton Fish on the Sickness and Mortality on Emigrant Ships, have been received and will be noticed in our next number.

---

*To Correspondents.*—The communication of Rev. C. L. Hequembourg, on the "Existence of an Interparietal Bone," is received and will appear in our next.

